# Mucosal immunization with an adenoviral vector vaccine confers superior protection against RSV compared to natural immunity

**DOI:** 10.3389/fimmu.2022.920256

**Published:** 2022-07-28

**Authors:** Clara Maier, Jana Fuchs, Pascal Irrgang, Michael Hermann Wißing, Jasmin Beyerlein, Matthias Tenbusch, Dennis Lapuente

**Affiliations:** ^1^ Institute of Clinical and Molecular Virology, University Hospital Erlangen, Friedrich-Alexander University Erlangen-Nürnberg, Erlangen, Germany; ^2^ Department of Molecular and Medical Virology, Ruhr University Bochum, Bochum, Germany

**Keywords:** RSV (respiratory syncytial virus), tissue-resident memory T cells, TRM, natural immunity, vaccine, mucosal, adenoviral (Ad) vector, IgA

## Abstract

Respiratory syncytial virus (RSV) infections are the leading cause of severe respiratory illness in early infancy. Although the majority of children and adults mount immune responses against RSV, recurrent infections are frequent throughout life. Humoral and cellular responses contribute to an effective immunity but also their localization at respiratory mucosae is increasingly recognized as an important factor. In the present study, we evaluate a mucosal vaccine based on an adenoviral vector encoding for the RSV fusion protein (Ad-F), and we investigate two genetic adjuvant candidates that encode for Interleukin (IL)-1β and IFN-β promoter stimulator I (IPS-1), respectively. While vaccination with Ad-F alone was immunogenic, the inclusion of Ad-IL-1β increased F-specific mucosal immunoglobulin A (IgA) and tissue-resident memory T cells (T_RM_). Consequently, immunization with Ad-F led to some control of virus replication upon RSV infection, but Ad-F+Ad-IL-1β was the most effective vaccine strategy in limiting viral load and weight loss. Subsequently, we compared the Ad-F+Ad-IL-1β-induced immunity with that provoked by a primary RSV infection. Systemic F-specific antibody responses were higher in immunized than in previously infected mice. However, the primary infection provoked glycoprotein G-specific antibodies as well eventually leading to similar neutralization titers in both groups. In contrast, mucosal antibody levels were low after infection, whereas mucosal immunization raised robust F-specific responses including IgA. Similarly, vaccination generated F-specific T_RM_ more efficiently compared to a primary RSV infection. Although the primary infection resulted in matrix protein 2 (M2)-specific T cells as well, they did not reach levels of F-specific immunity in the vaccinated group. Moreover, the infection-induced T cell response was less biased towards T_RM_ compared to vaccine-induced immunity. Finally, our vaccine candidate provided superior protection against RSV infection compared to a primary infection as indicated by reduced weight loss, virus replication, and tissue damage. In conclusion, our mucosal vaccine candidate Ad-F+Ad-IL-1β elicits stronger mucosal immune responses and a more effective protection against RSV infection than natural immunity generated by a previous infection. Harnessing mucosal immune responses by next-generation vaccines is therefore a promising option to establish effective RSV immunity and thereby tackle a major cause of infant hospitalization.

## Introduction

RSV is the leading cause of respiratory disease and hospitalization during early infancy. It is estimated that 33.8 million RSV-associated acute lower respiratory tract infections occur annually in children younger than five years leading to 66,000 to 199,000 deaths (1). By the age of two, almost all children were infected at least once with this virus. While the majority of infections result in mild disease, RSV bronchiolitis in early childhood has been associated with delayed respiratory sequelae such as wheezing cough or allergic sensitization in general (2). Although RSV-specific serum antibodies can be found in almost every child or adult, and RSV is relatively conserved, reinfections occur frequently throughout life (3). Especially in frail, elderly adults, such reinfections bear a risk for severe illness. 78% of all RSV-related deaths occur in elderly >65 years old (4). The reasons for this insufficient immunity after natural infections are incompletely understood. A short half-life of infection-induced humoral responses (5) and a generally weak induction of virus-specific antibodies (6) and T cells (7) by an RSV infection have been reported. This might be explained in part by a suppression of innate immune responses by several RSV proteins that in turn inhibits the establishment of adaptive memory responses (8).

The presence or absence of mucosal immune responses induced by previous RSV infections is increasingly recognized as a decisive factor for disease severity in humans (6, 9–11) and mice (12, 13) as also reported for other respiratory viruses like influenza A (14–17) or severe acute respiratory syndrome coronavirus 2 (18). Important components of adaptive mucosal immune responses are secretory IgA and T_RM_. Secretory IgA coats virus particles right after entering the respiratory tract and therefore likely provides an immediate virus neutralization. Moreover, its specific interaction with Fcα receptors might enable unique effector functions, whereas its polymeric form might increase antibody avidity (19). Mucosal IgA was reported to correlate with protection against RSV reinfections in humans (6, 9), but IgA levels seem to be poorly maintained in convalescents (6). Similar to these humoral immune parameters, T cells can persist in the respiratory tract as well. Those T_RM_ can easily be identified by their expression of CD69 and CD103, which is their prototypic phenotype in murine and human lung tissues (14, 15, 20, 21). A human challenge study with RSV followed pulmonary T_RM_ dynamics upon infection. It found a correlation of preexisting RSV-specific CD8^+^ T_RM_ and reduced clinical symptoms and viral load upon infection. Therefore, this study implies that T_RM_ provide protection against human RSV infections (11), although they are obviously not maintained at protective levels in all RSV-experienced individuals. Mouse models underline that RSV-induced T_RM_ responses provide protective immunity against secondary infections but wane substantially within five months (12, 13). Importantly, high levels of IgA correlate with protection against PCR-confirmed infection in humans but once an infection occurred do not influence the viral load (6). In contrast, airway T_RM_ might function as a second, complementary layer of immunity that cannot prevent infection but reduces virus replication upon infection (11).

Despite the disease and healthcare burden of RSV, prophylactic measures are still restricted to passive immunization with monoclonal antibodies. For a long time, vaccine research was largely affected by the unexpected outcome of an early vaccine trial in the late 1960s. A formalin-inactivated RSV vaccine given to toddlers and infants did not decrease the rate of community-acquired RSV infections but increased the rate of hospitalization from 5% in the control cohort to 80% in the vaccine group (22). Much effort has been spent on investigating the mechanisms behind this vaccine-enhanced RSV disease. Possible mechanisms include an induction of poorly neutralizing antibodies (nAbs) due to a disruption of important epitopes during the formalin inactivation (23–25) and a suboptimal Toll-like receptor stimulation upon immunization (26). Drivers of the vaccine-enhanced disease include the production of IL-4, IL-5, and IL-13 associated with T_H_2 responses, and eosinophilia (27, 28) upon infection. Although there is still no market approved vaccine against RSV available, several platforms including protein-based, nucleic acid-based, and viral vector vaccines are currently at different preclinical or clinical stages (29). One advanced candidate is Janssen’s adenoviral vector vaccine named Ad26.RSV.preF. It is based on the rare human adenovirus serotype 26 and encodes the RSV F protein in its stabilized prefusion conformation. In a human challenge study, the vaccine injected intramuscularly prevented about 40% of PCR-confirmed RSV infections compared to the placebo group four weeks after immunization (30) and is now tested in a phase III clinical trial (NCT04908683). Another vaccine candidate is Moderna’s mRNA-1345. The messenger RNA vaccine that also encodes for the stabilized prefusion F glycoprotein has recently entered phase III clinical trials (NCT05127434) after been granted a fast track designation by the U.S. Food and Drug Administration (FDA). Since both vaccine candidates are administrated intramuscularly, they predominantly provoke systemic immunity. Although repeated systemic immunizations can elicit low to moderate levels of humoral and cellular responses in the respiratory tract (31), a mucosal vaccination is more effective in this regard (32). Particularly the establishment of respiratory T_RM_ is highly dependent on a mucosal vaccine delivery as shown in different contexts (14, 15, 18). Overall, mucosal vaccines that generate local immunity in addition to systemic responses appear promising given the protective roles of mucosal immune responses in respiratory virus infections.

Here we describe a mucosal vaccination strategy based on an adenoviral serotype 5 vector expressing RSV F supplemented by vector-encoded IPS-1 (Ad-IPS-1) or IL-1β (Ad-IL-1β) as genetic adjuvants. Without an adjuvant, Ad-F resulted in moderate levels of mucosal immunity and reduced viral burden upon RSV infection, whereas the inclusion of Ad-IL-1β significantly increased the vaccine immunogenicity and protective efficacy. In direct comparison to natural immunity induced by a primary RSV infection, the vaccination with Ad-F+Ad-IL-1β raised stronger F-specific immune responses, particularly in the respiratory tract, and led to less virus replication and decreased weight loss upon an RSV infection. These results are encouraging indicating that an effective immunity against RSV is possible by taking advantage of mucosal vaccine strategies.

## Material and methods

### Adenoviral vaccines

The vaccine vector encoding RSV-F was constructed by insertion of the codon-optimized full-length sequence of the RSV-A2 F protein (GenBank database entry EF566942) as described before (33). The murine mature IL-1β-encoding vector and the Ad-empty control were constructed as described elsewhere (16). For the generation of Ad-IPS-1, the full-length cDNA sequence (NCBI Ref Seq NM_001206385.1) was initially cloned into the pShuttle vector. Replication-deficient adenoviral vectors were produced according to the AdEasy adenoviral vector system (34). Viral particles were purified and concentrated with the Vivapure Adenopack20 (Sartorius). The concentration of total Ad was measured by optical density at 260 nm (OD_260_) and infectious particles by Reed-and-Muench’s tissue-culture infectious dose 50 assay (35). Ratios of total to infectious particles usually were in the range of 200:1.

### Luciferase promoter reporter assays

In a 96-well plate, confluent HEK 293T cells were transfected with 0.2 μg of a luciferase reporter plasmid for promoter (binding) activity of either IFN (Interferon) -β, IRF3 (Interferon regulatory factor 3), NFκB (nuclear factor ‘kappa-light-chain-enhancer’ of activated B-cells), or ISRE (Interferon-sensitive response element), respectively (36). These plasmids are based on the pGL2-TATA-inr backbone (Stratagene) and contain the respective response elements/promoters that control the expression of firefly luciferase from *P. pyralis*. 24 hours later, the transfected cells were transduced with the replication-defective adenoviral vectors at a multiplicity of infection (MOI) of 10. After 48 hours incubation at 37˚C and 5% CO_2_, the cells were lysed with 100 μl Glo-lysis buffer, 25 μl Bright-Glo Luciferase substrate (both Promega) was added, and the signal (relative light units per second, RLU/s) was measured on a microplate luminometer (VICTOR X5, Perkin Elmer).

### Mice and immunizations

Six to eight-week-old BALB/cJRj mice were purchased from Janvier (Le Genest-Saint-Isle, France) and were kept in individually ventilated cages in accordance with German law and institutional guidelines under specific pathogen-free conditions, with constant temperature (20–24 °C) and humidity (45–65%) on a 12 h/12 h-light/dark cycle. Experimental and control animals were co-housed. The research staff was trained in animal care and handling in accordance to the FELASA and GV-SOLAS guidelines. Immunizations were conducted under general anesthesia (100 mg/kg ketamine and 15 mg/kg xylazine). A dose of 2x10^8^ particles (as defined by OD_260_) Ad-RSV-F and 1x10^9^ particles encoding for the adjuvants were given intranasally in a total volume of 50µl PBS (phosphate-buffered saline). Blood samples were taken on stated time points from the retroorbital sinus under light anesthesia with inhaled isoflurane. Bronchoalveolar lavages (BAL) were conducted after sacrificing the animals by cannulation of the trachea following two rinses with 1 ml PBS. Lungs and spleens were removed for T cell analyses.

### FACS-based antibody analysis

Target cells were either generated by stable lentiviral transduction of human embryonic kidney (HEK) 293A cells (for RSV F) or by transient transfection of HEK 293T cells (for RSV G; 11.5x10^6^ cells in a 175cm2 flask, 35 µg pCG-RSV, 17.5 µg blue-fluorescent protein, 78.75 µg polyethylenimine). For the assay, cells were harvested with a plastic scraper and 2x10^5^ cells were plated in a 96-well plate and were incubated with sera and BAL samples diluted in FACS-PBS (PBS with 0.5% BSA and 1 mM sodium azide) for 20 min at 4°C. F-specific antibodies were then detected with polyclonal anti-mouse Ig-FITC (1:300, 4 °C, 20 min incubation; BD Biosciences) or anti-mouse IgA-FITC (1:300, clone C10-3, BD Biosciences). Date were acquired on an AttuneNxT (ThermoFisher) and the mean fluorescence intensity (MFI) of the respective fluorescence signals were analyzed with FlowJoTM software (Treestar Inc.).

### Antigen-specific antibody ELISA

ELISA-plates were coated with 5x10^5^ plaque-forming units (PFU) heat-inactivated RSV-A2 in 100 μl carbonate buffer (50 mM carbonate/bicarbonate, pH 9.6) per well overnight at 4°C. Subsequently, the binding sites were blocked with 5% skimmed milk in PBS-T (PBS including 0.05% Tween-20) for one hour at room temperature and the plates were washed with PBS-T. Sera, diluted in 2% skimmed milk in PBS-T, were added to the wells. After 1h incubation at 4°C and three washing steps, secondary detection antibodies were added for 1h at RT: HRP-coupled anti-mouse Ig (1:1000, polyclonal, Daco), anti-mouse IgG1 (1:1000, clone X56, BD Biosciences), anti-mouse IgG2a (1:1000, clone R19-15, BD Biosciences), or anti-mouse IgA (1:5000, polyclonal, Bethyl Laboratories). Plates were washed seven times before ECL solution was added and the signal (relative light units per second, RLU/s) acquired on a microplate luminometer (VICTOR X5, PerkinElmer) using PerkinElmer 2030 Manager software.

### RSV microneutralization assay

As described before (33), twofold dilution series of complement-inactivated (56°C, 30 min) sera and BAL samples were incubated with 50 PFU rgRSV, a recombinant RSV-A2 strain expressing a green fluorescent protein, in a total volume of 100 µl Dulbecco’s modified Eagle’s medium (DMEM; no FCS, 2 mM L-Glutamine, 100 units/ml penicillin/streptomycin) for 1h at 37°C. Subsequently, 50 µl DMEM (1% FCS, 2 mM L-Glutamine, 100 units/ml penicillin/streptomycin) containing 10^4^ Hep2 cells were added per well and incubated for 72h at 37°C. Plaques were counted with an ELISPOT reader and analyzed using CTL Immunospot software (Cellular Technology limited BioSpot). 75% plaque reduction neutralization titers (PRNT75) were defined as the highest reciprocal serum/BAL dilution that inhibited more than 75% of plaques observed in infected control wells without serum/BAL treatment on the same plate (mean of four control wells per plate used).

### T cell assays

Where indicated, mice were injected with 3 µg anti-CD45-BV510 (clone 30-F11, Biolegend) intravenously and were euthanized 3 min later with an overdose of inhaled isoflurane to define circulatory and T_RM_. Lungs were cut in pieces and were digested in 2 ml R10 medium (RPMI 1640 supplemented with 10% FCS, 2 mM L-Glutamine, 10 mM HEPES, 50 μM β-mercaptoethanol and 1% penicillin/streptomycin) including 160 units DNase I and 500 units Collagenase D for 45 min at 37°C. Subsequently the digested lungs and spleens were homogenized through 70 µm cell strainers and treated with ammonium-chloride-potassium buffer to lyse erythrocytes. 10^6^ spleen cells or one fifth of the lung suspension were plated per well in 96-well plates for peptide restimulation and phenotypic analyses. For the restimulation assays, 100 µl R10 including monensin (2 µM), anti-CD28 (1 µg/ml, eBioscience), anti-CD107a-FITC (1:200, clone eBio1D4B; eBioscience) and respective MHC-I peptides; F CD8 peptides (F_80-94_ KQELDKYKNAVTELQ; F_84-98_ DKYKNAVTELQLLMQ; F_243-257_ DKYKNAVTELQLLMQ; F_247-261_ VSTYMLTNSELLSLI) or M CD8 peptide (M2_82-90_ SYIGSINNI) were added and incubated for 6h at 37°C. After the stimulation the samples were stained with anti-CD8a-Pacific blue (1:2000, clone 53-6.7, Biolegend), anti-CD4-PerCP (1:2000, clone RM4-5, eBioscience) and Fixable Viability Dye eFluor^®^ 780 (1:4000, eBioscience) in FACS-PBS for 20 min at 4°C. After fixation (2% paraformaldehyde, 20 min, 4°C) and permeabilization (0.5% saponin in FACS-PBS, 10 min, 4 °C), cells were stained intracellularly with anti-IL-2-APC (1:300, clone JES6-5H4, Biolegend), anti-TNF-PECy7 (1:300, clone MPG-XT22, Biolegend), and anti-IFNγ-PE (1:300, clone XMG1.2, Biolegend). The gating strategy is shown in **Supplementary Figure 3**. For phenotypic analyses, lymphocytes were stained with APC-labelled H-2K^D^ F_85-93_ (KYKNAVTEL) or APC-labelled H-2K^D^ M2_82-90_ (SYIGSINNI; both ProImmune) pentamers followed by a second staining step with anti-CD11a-eFluor450 (1:500, clone M17/4, Invitrogen), anti-CD8-BV711 (1:300, clone 53-6.7, BioLegend), anti-CD127-FITC (1:500, clone A7R34, BioLegend), anti-CD69-PerCP-Cy5.5 (1:300, clone H1.2F3, BioLegend), anti-CD103-PE (1:200, clone 2E7, eBioscience), and anti-KLRG1-PE-Cy7 (1:300, clone 2F1, eBioscience). The gating strategy is shown in **Supplementary Figure 2**. Data were acquired on an AttuneNxT (ThermoFisher) and analyzed using FlowJoTM software (Treestar Inc.).

### RSV infections

Experimental RSV infections were conducted under general anesthesia (100 mg/kg ketamine and 15 mg/kg xylazine). The inoculum containing 5x10^5^ PFU (primary RSV infection) or 1x10^6^ PFU (RSV protection experiments) RSV-A2 (ATCC VR-1540) was administered intranasaly in 50µl PBS. Animals were monitored daily for body weight and clinical score. In the challenge infections, mice were sacrificed five days post-infection. Lungs were harvested and homogenized in M-tubes with a GentleMACS dissociator (Miltenyi Biotec). Viral RNA was detected in cell-free supernatants of the lung homogenates *via* quantitative reverse transcriptase real-time PCR (qRT-PCR) using the GoTaq^®^ RT-qPCR 1-Step Kit (Promega) with the following primers for a sequence in the nucleoprotein gene: for agatcaacttctgtcatccagcaa; rev gcacatcataattaggagtatcaat). The lower limit of quantification was at 666 copies/ml. As correlate of tissue destruction, the total amount of protein in cell-free BAL samples was measured by a bicinchoninic acid assay (Pierce).

### Statistical analysis

Results are shown as mean ± SEM or as median ± interquartile range except it is described differently. Statistical analyses were performed with Prism 9.0 (GraphPad Software, Inc.). A p < 0.05 was considered statistically significant.

## Results

### Inclusion of Ad-IL-1β in the vaccine increases systemic and mucosal antibody responses

In the present study, we evaluate the immunogenicity and protective efficacy of an Ad5-based mucosal vaccine encoding for RSV F (Ad-F). Our previous study proved that the inclusion of inflammatory stimuli as genetic adjuvants can substantially increase the vaccine efficacy in mucosal immunizations (16). Therefore, we evaluated the use of vector-encoded IL-1β and IPS-1 (also known as mitochondrial antiviral-signaling protein, MAVS) as adjuvants in this part. Before using them *in vivo*, the genetic adjuvants were tested *in vitro* by luciferase reporter assays. Transduction of HEK 293T cells with Ad-IL-1β provoked a strong induction of the NFκB pathway, while Ad-IPS-1 additionally initiated a type I IFN response as seen by activation of the ISRE, IRF3, and IFN-β promotor reporters (**Supplementary Figure 1**).

**Figure 1:**
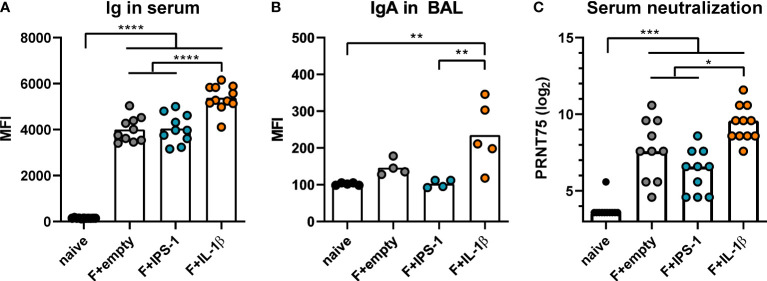
Vaccine-induced antibody responses. BALB/cJRj mice were immunized with 2x10^8^ particles Ad-F and 1x10^9^ particles Ad-empty, Ad-IPS-1, or Ad-IL-1β. Antibody levels were characterized in sera 35 days and in BAL samples 42 days post-immunization. **(A, B)** FACS-based analysis for F-specific antibodies was performed for Ig in sera **(A**, 1:100 dilution, detection with anti-Ig-FITC**)** and for IgA in BAL samples **(B**, 1:20 dilution, detection with anti-IgA-FITC**)** with HEK 293A cells expressing the F protein. Shown are the MFIs for each sample and the mean of the values for each group. **(C)** 75% plaque reduction neutralization titers (PRNT75) were analyzed in sera by RSV microneutralization assay (detection limit at a titer of <1:12). Bars represent group means **(A, B)** or medians **(C)** overlaid with individual data points. n=10-11 for sera and n=4-5 for BALs. Data were analyzed by one-way ANOVA followed by Tukey´s post test (values in C were log-transformed before analysis). *p<0.05, ** *p*<0.005, *** *p*<0.0005, **** *p*<0.0001.

Next, BALB/c mice were intranasally immunized with Ad-F plus the adjuvants or an empty vector construct (Ad-empty). Vaccine-induced antibody responses against RSV F were analyzed 35 days and 49 days later in serum and BAL samples, respectively. In a cytometry-based antibody detection method based on HEK 293A cells expressing the F protein in its natural conformation, all immunized groups presented robust F-specific antibody levels in sera (**Figure 1A**). The inclusion of Ad-IL-1β but not Ad-IPS-1 further increased antibody levels. Moreover, this adjuvant effect was not restricted to the systemic compartment but was also detectable in BAL samples that showed increased levels of F-specific IgA (**Figure 1B**). Correspondingly, all immunization strategies resulted in significant *in vitro* neutralization activity with the highest titers in the Ad-F+Ad-IL-1β group underlining its adjuvant properties (**Figure 1C**).

### Inclusion of Ad-IL-1β in the vaccine enhances mucosal T cell immunity

**Figure 2:**
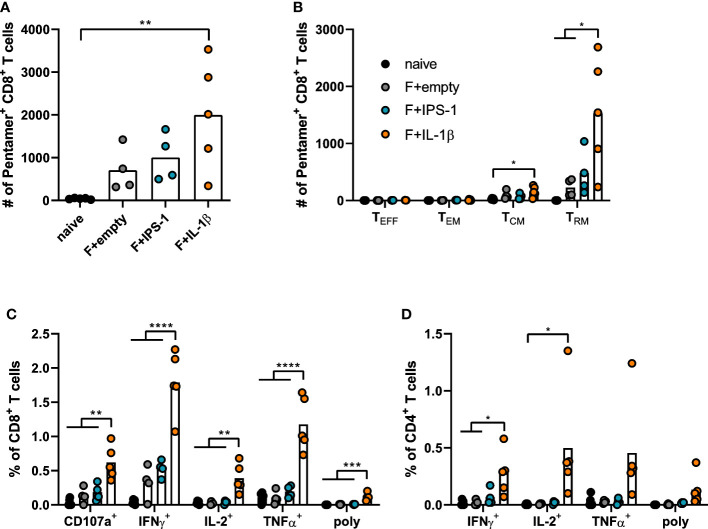
Vaccine-induced T cell responses in the lung. BALB/cJRj mice were immunized as described above and lung lymphocytes were analyzed 49 days later. Counts of F_85-93_ pentamer^+^ CD8^+^ T cells per lung **(A)** and counts of specific memory T cell subsets **(B)** are shown; effector T cells, T_EFF_, KLRG1^+^CD127^-^; effector memory T cells, T_EM_: KLRG1^+^CD127^+^; central memory T cells, T_CM_: KLRG1^-^CD69^-^CD103^-^CD127^+^; T_RM_: KLRG1^-^CD127^+/-^CD69^+^CD103^+^; gating scheme in **Supplementary Figure 2**. **(C, D)** Lung lymphocytes were restimulated with MHC-class I and class II peptides derived from the F protein and the functionality of CD8^+^ T cells **(C)** and CD4^+^ T cells **(D)** was detected *via* extracellular staining for CD107a and intracellular cytokine staining (poly; for CD8^+^: CD107a^+^IFNγ^+^IL-2^+^TNFα^+^; for CD4^+^: IFNγ^+^IL-2^+^TNFα^+^; gating strategy in **Supplementary Figure 3**). Bars represent group means overlaid with individual data points. n=4-5. Data were analyzed by one-way ANOVA followed by Tukey´s post Test. *p<0.05, ** *p*<0.005, *** *p*<0.0005, **** *p*<0.0001.

Seven weeks after the immunization, T cell responses were investigated in lymphocytes isolated from lung tissue. Using pentamer staining for F_85-93_-specific CD8^+^ T cells, it became evident that all immunizations established F-specific CD8^+^ T cells (**Figure 2A**). To further characterize those antigen-specific CD8^+^ T cells, we performed a phenotypic analysis to differentiate between different circulating memory T cell subsets (effector memory T cells, T_EM_: KLRG1^+^CD127^+^; central memory T cells, T_CM_: KLRG1^-^CD69^-^CD103^-^CD127^+^) and T_RM_ (KLRG1^-^CD127^+/-^CD69^+^CD103^+^; gating scheme in **Supplementary Figure 2**). As expected for a mucosal immunization route, most vaccine-induced, F_85-93_-specific CD8^+^ T cells in the lung were of a T_RM_ phenotype (**Figure 2B**). Substantially less T_CM_ and no T_EM_ or T_EFF_ were passaging through the lung at the time point of analysis. The vaccination with Ad-F+Ad-IL-1β increased lung CD8^+^ T_RM_ significantly compared to a non-adjuvanted vaccination with Ad-F+Ad-empty. Similarly, also the functional analyses of F-specific CD8^+^ (**Figure 2C**) and CD4^+^ T cell responses (**Figure 2D**) underlined the T cell stimulatory effect of Ad-IL-1β leading to substantial increases of cytokine-producing (IFNγ^+^, IL-2^+^, TNFα^+^) or degranulating (CD107a^+^) T cells upon peptide restimulation (**Supplementary Figure 3**). Polyfunctional CD8^+^ T cell populations positive for all assessed effector functions were elevated as well by the co-administration of Ad-IL-1β. In conclusion, a mucosal vaccination with Ad-F results in robust humoral and cellular immunity. The inclusion of Ad-IL-1β but not Ad-IPS-1 further increased systemic and mucosal immune responses.

### Ad-IL-1β-adjuvanted mucosal immunization increases protection against RSV infection

**Figure 3:**
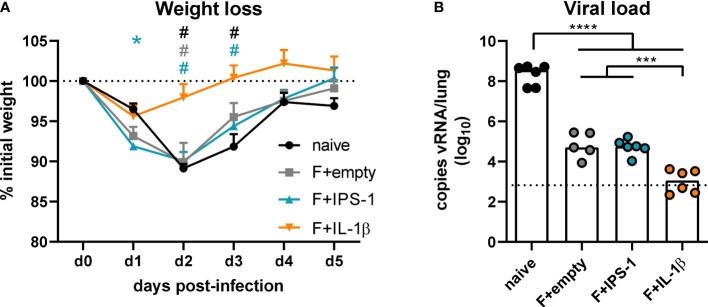
Vaccine efficacy against RSV infection. Mice were experimentally infected with 1x10^6^ PFU RSV-A2 48 days after the immunization. **(A)** The initial weight was set as 100% (dotted line) and weight changes were monitored for five days. **(B)** Viral RNA copies were quantified by qRT-PCR in lung homogenates five days post-infection. The dotted line represents the lower limit of quantification. Data represent group means with SEM **(A)** or group medians overlaid with individual data points **(B)**. n=5-6. Data were analyzed by one-way ANOVA followed by Tukey´s post test (values in B were log-transformed before analysis). For **(A)**: *p<0.05 *vs*. naive, ^#^ *p*<0.05 *vs*. F+IL-1β. For **(B)**: *p<0.05, ** *p*<0.005, *** *p*<0.0005, **** *p*<0.0001.

To assess the efficacy of the different vaccination strategies, mice were infected seven weeks after the immunization with RSV-A2. The unvaccinated group as well as the groups that received no adjuvant or Ad-IPS-1 showed a peak weight loss on day two post-infection followed by a regain of weight until day five (**Figure 3A**). In contrast, the group that received Ad-IL-1β presented only a minor weight loss on day one and then started to recover reaching its initial weight about two days earlier than the other vaccinated groups. Viral load in the lung was investigated five days post-infection by qRT-PCR detection of viral RNA. Naïve animals showed the most pronounced virus replication, whereas all vaccine regimens reduced viral load at least by a factor of 1000 on day 5 (**Figure 3B**). In line with the enhanced immune responses provoked by the inclusion of Ad-IL-1β in the vaccination, animals of this group had significantly lower viral loads compared to the ones of the other immunization groups.

### Mucosal immunization generates superior mucosal antibody responses compared to natural infection

**Figure 4:**
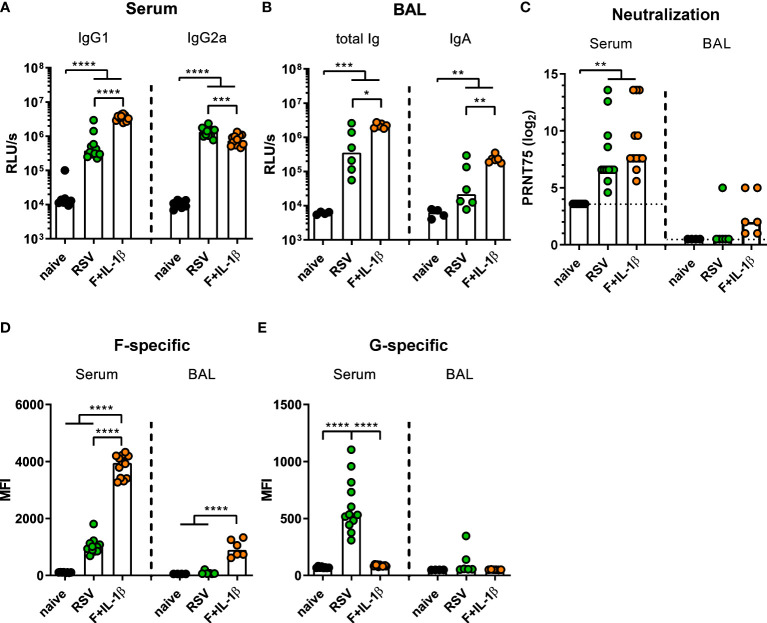
Humoral responses induced by mucosal vaccination or primary RSV infection. BALB/cJRj mice were infected with 5x10^5^ PFU RSV-A2 or immunized with 2x10^8^ particles Ad-F and 1x10^9^ particles Ad-IL-1β. Antibody levels were characterized in sera 35 days and in BAL samples 42 days post-immunization. **(A, B)** Virus-specific IgG1 and IgG2a in sera **(A**, 1:1000 dilution**)** and Ig and IgA in BAL **(B**, 1:10 dilution**)** were detected *via* virus-coated ELISA. **(C)** 75% plaque reduction neutralization titers (PRNT75) in sera (detection limit at a titer of <1:12, dotted line) and BAL (detection limit at a titer of <1:2, dotted line) were tested by microneutralization assay. **(D, E)** FACS-based analysis for F- **(D)** and G-specific **(E)** antibodies was performed in sera (1:100 dilution) and BAL samples (1:20 dilution). Bars represent group medians **(A-C)** or means **(D, E)** overlaid with individual data points. n=8-12 for sera and n=4-6 for BALs. Data were analyzed by one-way ANOVA followed by Tukey´s post test (values in A-C were log-transformed before analysis). *p<0.05, ** *p*<0.005, *** *p*<0.0005, **** *p*<0.0001. Relative light units per second, RLU/s.

Next, we compared the immune responses raised by a natural RSV infection with the ones induced by our mucosal vaccine candidate Ad-F+Ad-IL-1β. To this end, mice were immunized as described above or were infected with RSV-A2. Five weeks (serum) and seven weeks (BALs) later humoral immune responses were assessed. In order to capture all RSV-specific antibody moieties, we performed ELISA analyses with coated, heat-inactivated RSV-A2. Humoral responses in sera showed a bias towards IgG1 after mucosal immunization compared to a more IgG2a-prone response after RSV infection (**Figure 4A**). Importantly, BAL samples contained about 10-fold higher levels of RSV-specific antibodies of all isotypes (**Figure 4B** left) and secretory IgA (**Figure 4B** right) after adenoviral immunization compared to the RSV-immune group. Serum neutralization titers were comparable in the RSV-immune and Ad-F+Ad-IL-1β groups (**Figure 4C**), while neutralization in BAL samples was overall low. However, there was a trend that all mucosally vaccinated mice mounted a low-level virus neutralization (6/6 mice), whereas in the previously infected group only one mouse showed detectable a neutralization titer (1/6 mice; **Figure 4C**).

While the adenoviral vaccination encodes only for one RSV protein, the primary infection probably generates humoral immunity against other RSV proteins as well. Therefore, the flow cytometric assay was used to determine the specific antibody levels against the two major surface proteins F and G. It was observed that F-specific serum antibodies were much more abundant in mucosally vaccinated mice compared to RSV-immune mice (**Figure 4D**). This was also evident in BAL samples, where the infection resulted in low to undetectable levels of F-specific antibodies, whereas the immunization provoked a response in all animals. However, the primary infection additionally induced G-specific humoral responses in sera, which were of course absent after Ad-F-Ad-IL-1β immunization (**Figure 4E**). Again, the RSV-immune group presented low to undetectable antibody levels in BAL samples. In conclusion, systemic antibody responses are quantitatively similar in both immune groups resulting in a comparable neutralization, but there are clear qualitative differences. Moreover, a mucosal immunization with our candidate vaccine is superior in inducing mucosal antibodies than a primary RSV infection.

### Mucosal vaccination but not natural infection establishes robust F-specific T_RM_ responses

**Figure 5:**
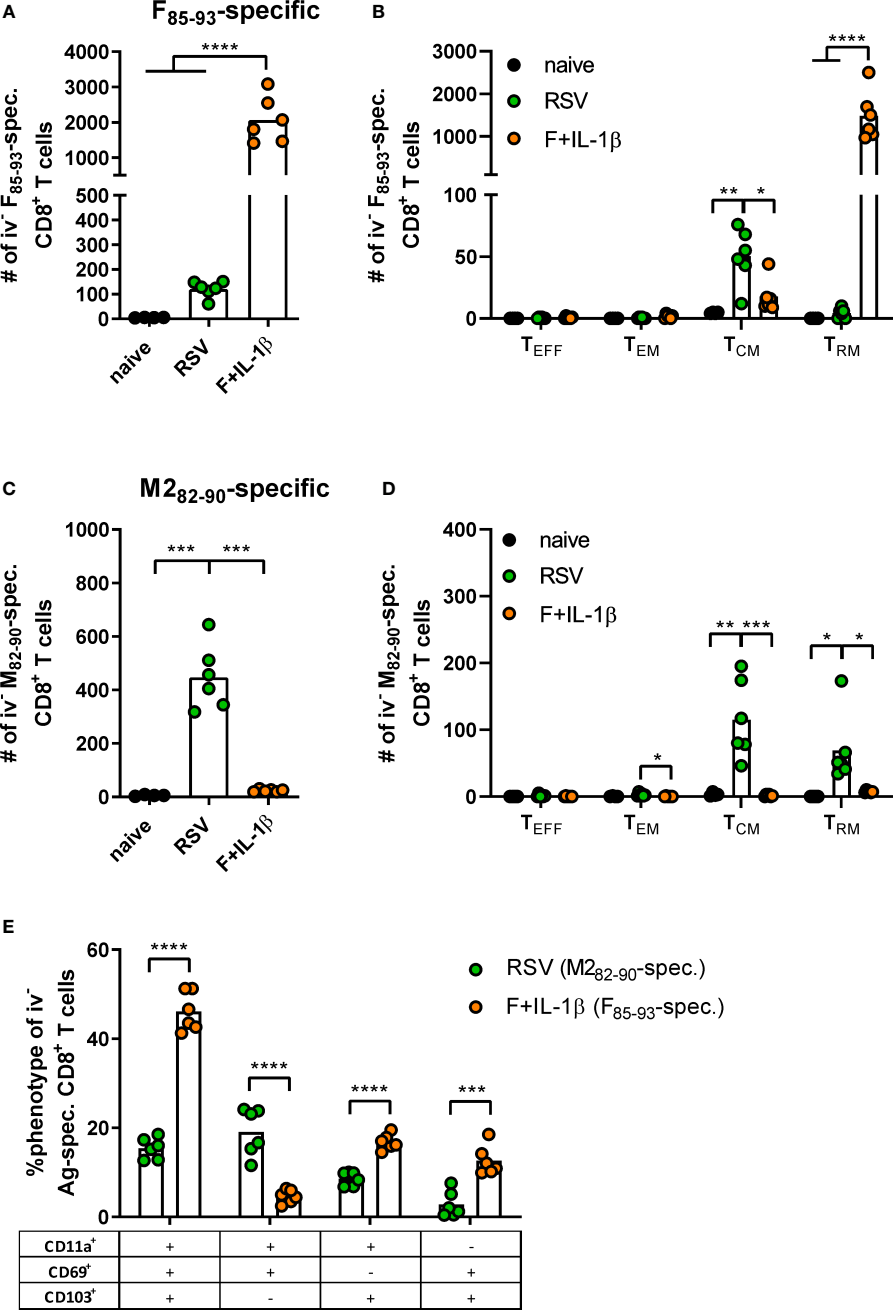
T cell phenotypes induced by mucosal vaccination and a primary RSV infection. BALB/cJRj mice were immunized or infected as described above and isolated lung lymphocytes were analyzed seven weeks later. Counts of F_85-93_ pentamer^+^ **(A)** and M2_82-90_ pentamer^+^ CD8^+^ T cells **(C)** per lung and of specific memory T cell subsets **(B, D)** are shown; effector T cells, T_EFF_, KLRG1^+^CD127^-^; effector memory T cells, T_EM_, KLRG1^+^CD127^+^; central memory T cells, T_CM_, KLRG1^-^CD69^-^CD103^-^CD127^+^; T_RM_, KLRG1^-^CD127^+/-^CD69^+^CD103^+^; gating scheme in **Supplementary Figure 2**. **(E)** Pentamer^+^ CD8^+^ T cells (M2_82-90_-spec. in RSV group, F_85-93_-spec. in Ad-F+Ad-IL-1β group) that were not stained by the intravenous staining were further characterized for their expression of CD11a, CD69, and CD103. Bars represent group means overlaid with individual data points. n=4-6. Data were analyzed by one-way ANOVA followed by Tukey´s post test **(A-D)** or two-tailed Student’s *t*-test **(E)**. *p<0.05, ** *p*<0.005, *** *p*<0.0005, **** *p*<0.0001.

**Figure 6:**
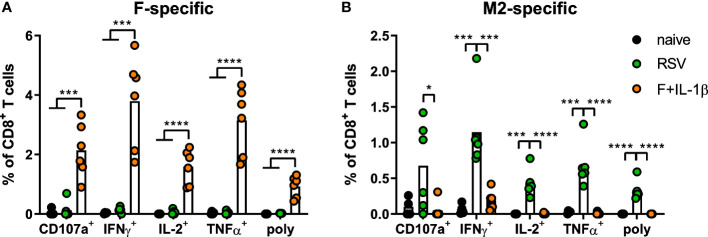
Mucosal T cell functionality induced by mucosal vaccination and a primary RSV infection. Lung lymphocytes were restimulated with MHC-class I peptides derived from the F **(A)** and the M2 protein **(B)** and the functionality of CD8^+^ T cells was detected *via* extracellular staining for CD107a and intracellular cytokine staining (poly; CD107a^+^IFNγ^+^IL-2^+^TNFα^+^; gating strategy in **Supplementary Figure 3**). Bars represent group means overlaid with individual data points. n=4-6. Data were analyzed by one-way ANOVA followed by Tukey´s post Test. *p<0.05, ** *p*<0.005, *** *p*<0.0005, **** *p*<0.0001.

As already shown above, our mucosal vaccine candidate elicits robust F_85-93_-specific CD8^+^ T cell responses in the lung (**Figure 5A**; **Figure 2**). In contrast to that, a primary RSV infection results in a 20-fold weaker F-specific response (**Figure 5A**). By using a combination of our phenotyping analysis and intravascular staining to distinguish circulating and resident memory T-cells (37), the vast majority of vaccine-induced, F-specific T cells was identified as T_RM_ as already pointed out earlier (**Figure 5B**; **Figure 2**). A primary infection was not very efficient in inducing T cells of that specificity and, interestingly, those few F-specific T cells show predominantly a T_CM_ phenotype. CD8^+^ T cell responses against another dominant epitope, M2_82-90_, were about fourfold stronger compared to F_85-93_-specific responses in the RSV-immune group, but do not reach the absolute level of F-specific T cell immunity observed after immunization (**Figure 5C**). Again, there was no clear dominance of T_RM_ over circulatory phenotypes as seen after mucosal Ad-F+Ad-IL-1β vaccination (**Figure 5D**) yielding roughly equal amounts of T_CM_ and CD69^+^CD103^+^ T_RM_ sampled in the lung. In order to compare T_RM_ phenotypes in a broader fashion independent of the strict CD69^+^CD103^+^ phenotype, we analyzed M2_82-90_-specific (RSV-immune) and F_85-93_-specific (mucosal immunization) CD8^+^ T cells in the lung that were negative for the intravascular staining. These T cells are per definition tissue-resident but also include populations aside from the most stringent CD69^+^CD103^+^ lung T_RM_ phenotype. By this approach, we could again prove that CD69^+^CD103^+^ T_RM_ are indeed the dominant lung-resident phenotype after mucosal vaccination and that most of these cells express CD11a^+^ as well (**Figure 5E**). However, such phenotypic dominance was absent after RSV infection. The primary infection mainly resulted in CD11a^+^ T_RM_ subsets with variable expression of CD69^+^ and/or CD103^+^. Of note, the frequencies of all subsets that express CD103^+^ are higher in the Ad-F+Ad-IL-1β group compared to the RSV-immune animals. To analyze CD8^+^ T cell functionality, lung lymphocytes were restimulated ex vivo with F- and M2-derived peptides. These analyses confirmed a strong F-specific response after mucosal vaccination, while a primary RSV infection did not raise reliable amounts of F-reactive T cells (**Figure 6A**). As expected from the multimer staining, moderate levels of M2-reactive CD8^+^ T cells were detectable in RSV-immune mice (**Figure 6B**). Similar trends were observed in the systemic T cell response as well (**Supplementary Figure 4**). Thus, a natural infection and our mucosal vaccination strategy led to very different T cell responses regarding amplitude, specificity, and memory phenotype. Substantial numbers of mucosal, F-specific T cells are not part of a naturally induced immunity in mice but can be established by a mucosal vaccination.

### Mucosal immunization establishes more efficient protection than natural immunity

**Figure 7:**
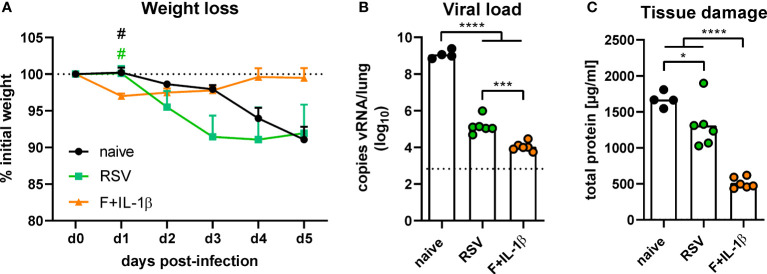
Protection against RSV infection mediated by vaccination and a primary RSV infection. Mice were experimentally infected with 1x10^6^ PFU RSV-A2 48 days after the immunization or primary infection. **(A)** The initial weight was set as 100% (dotted line) and weight changes were monitored for five days. **(B)** Viral RNA copies were quantified by qRT-PCR in lung homogenates five days post-infection. The dotted line represents the lower limit of quantification. **(C)** Tissue damage was assessed by measuring the total concentration of protein in BAL samples *via* bicinchoninic acid assay. Data represent group means with SEM **(A)**, group medians overlaid with individual data points **(B)**, or group means overlaid with individual data points **(C)**. n=4-6. Data were analyzed by one-way ANOVA followed by Tukey´s post test (values in B were log-transformed before analysis). For **(A)**: ^#^ *p*<0.05 *vs*. F+IL-1β. For **(B, C)**: *p<0.05, ** *p*<0.005, *** *p*<0.0005, **** *p*<0.0001.

The protective effects of natural immunity and the mucosal vaccination were then tested by an experimental infection with RSV-A2. Vaccinated mice showed a transient weight loss of ~3% between one and three days post-infection but started to regain weight already on day one reaching the initial weight on day four (**Figure 7A**). In contrast, RSV-immune and naïve mice started to lose weight on day two with a constant decrease until the end of the experiment on day five. There was even a trend towards more pronounced weight loss in the RSV-immune group compared to naïve mice, but this trend lacks statistical power (*p*=0.10 naïve *vs*. RSV at day three post-infection; one-way ANOVA followed by Tukey´s post test). Nevertheless, virus RNA levels in the lung were significantly reduced in presence of natural immunity compared to naïve animals (**Figure 7B**). In line with the lower weight loss, the adenoviral immunization resulted in the lowest viral RNA levels at day 5 after infection. As a surrogate of tissue damage, we measured the amount of total protein in BAL samples. Both immune groups showed a significant reduction of the protein concentration compared to naïve mice, but the RSV group presented a relatively high tissue damage compared to the vaccine group (**Figure 7C**). Therefore, although both a primary infection and a mucosal immunization reduce virus replication upon an RSV infection, the mucosal immunization provides a superior protection against weight loss and tissue damage.

## Discussion

RSV vaccine research was hampered by the severe complications observed during early clinical trials with a formalin-inactivated vaccine candidate in the 1960s. However, vaccine technologies have diversified and improved significantly in the last decades yielding several promising vaccine candidates that are currently FDA fast-tracked and/or entering phase III clinical trials (38). Importantly, none of these vaccines aims at the induction of mucosal immunity, although preclinical and clinical data suggest an important contribution of local immune responses to the control of RSV infections.

In the present study, we assessed the immunogenicity and efficacy of a mucosally administrated, adenoviral vaccine that encodes the wildtype RSV-A2 F protein. In the first part, we investigated whether the inclusion of genetic adjuvants increases vaccine-induced immunity. Two inflammatory proteins were chosen as vector-encoded adjuvants: IL-1β and IPS-1. IL-1β is a hallmark cytokine of tissue inflammation (39, 40) and particularly connected to T cell stimulating properties (41–43). Therefore, this cytokine was already used in several studies as adjuvant for example as mucosal adjuvant in flu vaccinations (16, 44) or as T cell stimulus in cancer vaccines (45–47). IPS-1 was chosen as adjuvant candidate since the retinoic acid inducible gene I (RIG-I)-IPS-1 pathway plays an important role in innate immune sensing of viral infections including RSV (48–50) and the subsequent activation of adaptive immune responses (51, 52) as exploited by the co-application of RIG-I agonists in vaccination settings (53, 54). Moreover, IPS-1 activation has been reported as essential for mounting T cell responses upon RSV reinfection (55) and specifically for the induction of mucosal RSV immunity (56).

In general, the mucosal administration of Ad-F showed a robust immunogenicity inducing F-specific antibodies and T cells. Mucosal immune responses including BAL IgA and CD8^+^ T_RM_ were rather low without adjuvantation, but the inclusion of Ad-IL-1β significantly increased those responses. Concomitantly, the protection against an RSV infection was enhanced as well indicated by reduced weight loss and virus replication upon infection. These observations are reminiscent of our previous studies in an influenza A model (16). Ad-IL-1β increased mucosal immunity in these experiments by activation of key steps in the establishment of T_RM_: mucosal inflammation, attraction of innate immune cells, increased pulmonary infiltration of T_RM_ precursor cells, and finally the upregulation of local factors that support *in situ* differentiation into CD69^+^CD103^+^ T_RM_ cells. The data generated in the present study underline that the adjuvant activities of IL-1β benefit mucosal vaccinations against different respiratory pathogens. In contrast, Ad-IPS-1 did not enhance vaccine immunogenicity. Although it clearly demonstrated strong activation of NFκB and the type I IFN pathway *in vitro*, none of the measured immune parameters was increased by the inclusion of Ad-IPS-1 in the mucosal vaccine. This was unexpected, since IPS-1-induced type I IFNs contribute to RSV-specific T_RM_ establishment (56). However, a defective CD8^+^ T_RM_ response in IPS-1 knockout mice might not directly be translatable into an enhanced response upon *in vivo* overexpression of IPS-1, and there also exists a contradictory report about the role of IPS-1 in RSV memory T cell formation (49). Moreover, murine type I IFNs consist of IFN-β and 14 IFN-α subtypes that differ in their immunomodulatory effects on CD8^+^ T cells (57). One cannot exclude that the vector-driven overexpression of IPS-1 results in a type I IFN profile that does not efficiently stimulate T and B cell responses.

After proving that Ad-F+Ad-IL-1β is an effective vaccine candidate that establishes systemic and mucosal immunity, we compared its immunogenicity profile and protective efficacy to natural immunity generated by a primary RSV infection. This is particularly important because natural immunity is obviously not sufficient to promote long-lived and efficient immunity against reinfections. Therefore, vaccine candidates should preferably induce stronger immune responses compared to an RSV infection. Most preclinical vaccine candidates and all vaccine approaches that entered phase III clinical trials aim at the induction of F-specific immunity. One important consideration for this choice is that the F protein is highly conserved and a major target for nAbs. The attachment protein G also raises nAbs, but it shows the highest variability among all RSV proteins suggesting that G-specific immunity is less cross-reactive (58). Our mucosal vaccine candidate Ad-F+Ad-IL-1β and a primary RSV infection both elicit robust RSV-specific antibody responses in serum. However, F-specific responses were substantially stronger after vaccination compared to a primary RSV infection. In contrast, the latter one raised G-specific antibodies as well probably explaining why both immunizations resulted in comparable virus neutralization by serum samples. Indeed, G-specific virus neutralization is commonly induced by a primary RSV infection in infants (59). Interestingly, the mucosal vaccination provoked robust levels of F-specific antibodies in BAL fluids, while F- and G-specific humoral responses were rather low or undetectable in BAL samples of previously infected mice. F-specific IgA is an important correlate of protection against RSV in non-human primates (60) and in a human challenge study (6). The latter study reports an impaired induction of IgA^+^ memory B cells after RSV infection, which is in line with our observations.

There is emerging evidence that mucosal CD4^+^ and CD8^+^ T cells contribute to a rapid and effective clearance of pulmonary pathogens with influenza A being the most investigated, prototypical example (14–16, 61). The Varga lab dissected the role of T cell responses in RSV infections in a series of publications. In conclusion, they have demonstrated that systemic CD8^+^ T cells alone mediate severe immunopathology upon infection in mice (62), which is prevented in presence of RSV-specific nAbs (63). The importance and protective capacity of lung T_RM_ against RSV was also underlined by works of this (13) and another group (12). The clinical relevance is highlighted by human challenge studies conducted by the group around Christopher Chiu and Peter Openshaw. In two studies they report on CD4^+^ and CD8^+^ T_RM_ dynamics upon human RSV infection and show that CD8^+^ T_RM_ abundance prior to infection correlates with reduced symptoms and virus replication (10, 11). Importantly, our present study proves that a natural RSV infection does not generate substantial T_RM_ responses against the F protein, while inducing moderate levels of M2-specific T_RM_ cells. Additionally, the infection-induced T cell response is not skewed towards mucosal T cell phenotypes but instead established similar levels of T_RM_ and T_CM_ as sampled in the lung. It is tempting to speculate that such a suboptimal generation of mucosal memory T cell responses is one reason for frequent RSV reinfections despite previous encounters with the pathogen. In contrast, our candidate vaccine elicits robust F-specific T_RM_ responses in the lung. Moreover, comparing the phenotype of infection- and immunization-induced T_RM_, the adenoviral vaccine raised a higher frequency of CD103^+^ T_RM_ cells. This might be connected to the upregulation of transforming growth factor β (TGF-β) by Ad-IL-1β upon immunization (16) and could promote enhanced cytotoxic T cell functions (64). In accordance with this enhanced immunogenicity, our mucosal vaccination strategy led to reduced weight loss, virus replication in the lung, and tissue damage upon infection. Importantly, although virus replication was also decreased in RSV-immune mice, they experienced a similar or even increased weight loss compared to naïve animals and a relatively high tissue damage. One might speculate that the impaired induction of mucosal immune responses in connection with moderate levels of circulating T cell responses lead to a pronounced immunopathology but this needs further investigation. Nevertheless, these data underline that natural RSV immunity is suboptimal, especially regarding mucosal, F-specific IgA and T_RM_. At the same time, we could show that a single, mucosal administration of Ad-F+Ad-IL-1β efficiently establishes these promising correlates of protection leading to an improved control of RSV infections.

Although the present study provides valuable insights into infection- versus mucosal immunization-induced immunity, these insights need confirmation in other models and settings as well. The BALB/c mouse model is commonly used as preclinical animal model and enabled important insights in RSV-related disease and immunopathology, but there are limitations (65). One is the semi-permissiveness of mice for RSV and therefore a relatively high inoculation dose is needed to achieve virus replication in the respiratory tract (66). The extent of virus replication might influence the resulting immune responses after an RSV infection. Moreover, clinical symptoms are only mild in murine RSV infections despite the high infectious doses. Weight loss upon an RSV infection, a commonly used disease progression marker in murine respiratory infections, is very mild and sometimes varies among different infection studies. Nevertheless, our data hind towards a defective generation of mucosal immunity upon RSV infection. It has to be investigated whether this is a general immune escape feature of RSV or a species-related phenomenon. However, the findings reported here fit well to data from clinical studies investigating RSV-specific immune responses after infection. Since most animal models have limitations, clinical studies with human volunteers remain the most meaningful setting. In particular, small-sized human challenge studies are well suited to investigate correlates of protection and vaccine efficacy, but are extremely difficult to conduct due to ethical and economic reasons.

## Data availability statement

The original contributions presented in the study are included in the article/**supplementary material**. Further inquiries can be directed to the corresponding author/s.

## Ethics statement

This study was approved by the Government of Lower Franconia, which nominated an external ethics committee that authorized the experiments. Studies were performed under the project license 55.2-2532-2-906 and 55.2.2-2532-2-1085.

## Author contributions

MT and DL designed and conceived the study. CM, JF, PI, MW, JB and DL conducted the experiments. CM, MT and DL analyzed the data and wrote the manuscript. All authors contributed to the article and approved the submitted version.

## Funding

The study was supported by funding from the German Research Foundation (DFG, TE1001/4-1, 419013006) and the Doktor-Robert-Pfleger Foundation.

## Acknowledgments

The present work was performed in fulfillment of the requirements for obtaining the degree “Dr. med.”

## Conflict of interest

The authors declare that the research was conducted in the absence of any commercial or financial relationships that could be construed as a potential conflict of interest.

## Publisher’s note

All claims expressed in this article are solely those of the authors and do not necessarily represent those of their affiliated organizations, or those of the publisher, the editors and the reviewers. Any product that may be evaluated in this article, or claim that may be made by its manufacturer, is not guaranteed or endorsed by the publisher.

## Supplementary material

Supplementary Figure 1 Bioactivity of genetic adjuvants. The activation of NFκB- **(A)**, ISRE- **(B)**, IRF3- **(C)** and IFN-β- **(D)** pathways were assessed by *in vitro* luciferase reporter assays. To this end, HEK 293T cells were transfected with respective luciferase reporter plasmid followed by a transduction with Ad-empty, Ad-IPS-1 or Ad-IL-1β. Transfected cells without transduction were used as negative control (no Ad). Data represent medians of technical triplicates with individual data points. Data were analyzed by one-way ANOVA followed by Tukey´s post test (values were log-transformed before analysis). **p*<0.05, ** *p*<0.005, *** *p*<0.0005, **** *p*<0.0001. Relative light units per second, RLU/s.

Supplementary Figure 2 Gating strategy for memory T cell phenotypes. Exemplary gating strategy of a lung sample from an Ad-F+Ad-IL-1β-immunized mouse. T_RM_ phenotypes are determined within the CD45-BV510 (intravascular staining) negative compartment, while circulatory T_EFF_, T_EM_, and T_CM_ are identified within the iv-positive compartment. T_EFF_, effector T cells; T_EM_, effector memory T cells; T_CM_, central memory T cells; T_RM_, tissue-resident memory T cells

Supplementary Figure 3 Gating strategy for the analysis of functional T cell responses. Exemplary gating strategy of a lung sample from an Ad-F+Ad-IL-1β-immunized mouse. The degranulation marker CD107 was only assessed in CD8^+^ T cells.

Supplementary Figure 4 Spleen T cell functionality induced by mucosal vaccination and a primary RSV infection. Spleen lymphocytes were restimulated with MHC-class I peptides derived from the F **(A)** and the M2 protein **(B)** and the functionality of CD8^+^ T cells was detected *via* extracellular staining for CD107a and intracellular cytokine staining (poly; for CD8^+^: CD107a^+^IFNγ^+^IL-2^+^TNFα^+^; gating strategy in **Supplementary Figure 3**). Bars represent group means overlaid with individual data points. n=4-6. Data were analyzed by one-way ANOVA followed by Tukey´s post Test. **p*<0.05, ** *p*<0.005, *** *p*<0.0005, **** *p*<0.0001.

## References

[1] Nair H Nokes DJ Gessner BD Dherani M Madhi SA Singleton RJ. Global burden of acute lower respiratory infections due to respiratory syncytial virus in young children: a systematic review and meta-analysis. Lancet (2010) 375:1545–55. doi: 10.1016/S0140-6736(10)60206-1 PMC2864404 20399493

[2] Sigurs N Aljassim F Kjellman B Robinson PD Sigurbergsson F Bjarnason R. Asthma and allergy patterns over 18 years after severe RSV bronchiolitis in the first year of life. Thorax (2010) 65:1045–52. doi: 10.1136/thx.2009.121582 20581410

[3] Hall CB Walsh EE Long CE Schnabel KC. Immunity to and frequency of reinfection with respiratory syncytial virus. J Infect Dis (1991) 163:693–8. doi: 10.1093/infdis/163.4.693 2010624

[4] Thompson WW Shay DK Weintraub E Brammer L Cox N Anderson LJ. Mortality associated with influenza and respiratory syncytial virus in the united states. JAMA (2003) 289:179. doi: 10.1001/jama.289.2.179 12517228

[5] Blunck BN Aideyan L Ye X Avadhanula V Ferlic-Stark L Zechiedrich L. A prospective surveillance study on the kinetics of the humoral immune response to the respiratory syncytial virus fusion protein in adults in Houston, Texas. Vaccine (2021) 39:1248–56. doi: 10.1016/j.vaccine.2021.01.045 PMC8152364 33509697

[6] Habibi MS Jozwik A Makris S Dunning J Paras A DeVincenzo JP. Impaired antibody-mediated protection and defective IgA b-cell memory in experimental infection of adults with respiratory syncytial virus. Am J Respir Crit Care Med (2015) 191:1040–9. doi: 10.1164/rccm.201412-2256OC PMC4435460 25730467

[7] Bont L Versteegh J Swelsen WTN Heijnen CJ Kavelaars A Brus F. Natural reinfection with respiratory syncytial virus does not boost virus-specific T-cell immunity. Pediatr Res (2002) 52:363–7. doi: 10.1203/00006450-200209000-00009 12193668

[8] Van Royen T Rossey I Sedeyn K Schepens B Saelens X. How RSV proteins join forces to overcome the host innate immune response. Viruses (2022) 14:419. doi: 10.3390/v14020419 35216012 PMC8874859

[9] Walsh EE Falsey AR. Humoral and mucosal immunity in protection from natural respiratory syncytial virus infection in adults. J Infect Dis (2004) 190:373–8. doi: 10.1086/421524 15216475

[10] Guvenel A Jozwik A Ascough S Ung SK Paterson S Kalyan M. Epitope-specific airway-resident CD4+ T cell dynamics during experimental human RSV infection. J Clin Invest (2019) 130:523–38. doi: 10.1172/JCI131696 PMC6934186 31815739

[11] Jozwik A Habibi MS Paras A Zhu J Guvenel A Dhariwal J. RSV-Specific airway resident memory CD8+ T cells and differential disease severity after experimental human infection. Nat Commun (2015) 6:10224. doi: 10.1038/ncomms10224 26687547 PMC4703893

[12] Kinnear E Lambert L McDonald JU Cheeseman HM Caproni LJ Tregoning JS. Airway T cells protect against RSV infection in the absence of antibody. Mucosal Immunol (2018) 11:249–56. doi: 10.1038/mi.2017.46 28537249

[13] Luangrath MA Schmidt ME Hartwig SM Varga SM. Tissue-resident memory T cells in the lungs protect against acute respiratory syncytial virus infection. ImmunoHorizons (2021) 5:59–69. doi: 10.4049/immunohorizons.2000067 33536235 PMC8299542

[14] Wu T Hu Y Lee Y-T Bouchard KR Benechet A Khanna K. Lung-resident memory CD8 T cells (TRM) are indispensable for optimal cross-protection against pulmonary virus infection. J Leukoc Biol (2014) 95:215–24. doi: 10.1189/jlb.0313180 PMC3896663 24006506

[15] Zens KD Chen JK Farber DL. Vaccine-generated lung tissue–resident memory T cells provide heterosubtypic protection to influenza infection. JCI Insight (2016) 1. doi: 10.1172/jci.insight.85832 PMC4959801 27468427

[16] Lapuente D Storcksdieck Genannt Bonsmann M Maaske A Stab V Heinecke V Watzstedt K. IL-1β as mucosal vaccine adjuvant: the specific induction of tissue-resident memory T cells improves the heterosubtypic immunity against influenza a viruses. Mucosal Immunol (2018) 11:1265–78. doi: 10.1038/s41385-018-0017-4 29545648

[17] Lapuente D Ruzsics Z Thirion C Tenbusch M. Evaluation of adenovirus 19a as a novel vector for mucosal vaccination against influenza a viruses. Vaccine (2018) 36:2712–20. doi: 10.1016/j.vaccine.2018.02.075 29628150

[18] Lapuente D Fuchs J Willar J Vieira Antão A Eberlein V Uhlig N. Protective mucosal immunity against SARS-CoV-2 after heterologous systemic prime-mucosal boost immunization. Nat Commun (2021) 12:6871. doi: 10.1038/s41467-021-27063-4 34836955 PMC8626513

[19] Terauchi Y Sano K Ainai A Saito S Taga Y Ogawa-Goto K. IgA polymerization contributes to efficient virus neutralization on human upper respiratory mucosa after intranasal inactivated influenza vaccine administration. Hum Vaccin Immunother (2018) 14:1351–61. doi: 10.1080/21645515.2018.1438791 PMC6037454 29425074

[20] Kumar BV Ma W Miron M Granot T Guyer RS Carpenter DJ. Human tissue-resident memory T cells are defined by core transcriptional and functional signatures in lymphoid and mucosal sites. Cell Rep (2017) 20:2921–34. doi: 10.1016/j.celrep.2017.08.078 PMC5646692 28930685

[21] Snyder ME Finlayson MO Connors TJ Dogra P Senda T Bush E. Generation and persistence of human tissue-resident memory T cells in lung transplantation. Sci Immunol (2019) 4:eaav5581. doi: 10.1126/sciimmunol.aav5581 30850393 PMC6435356

[22] Kim HW Canchola JG Brandt CD Pyles G Chanock RM Jensen K. Respiratory syncytial virus disease in infants despite prior administration of antigenic inactivated vaccine. Am J Epidemiol (1969) 89:422–34. 10.1093/oxfordjournals.aje.a120955 4305198

[23] Moghaddam A Olszewska W Wang B Tregoning JS Helson R Sattentau QJ. A potential molecular mechanism for hypersensitivity caused by formalin-inactivated vaccines. Nat Med (2006) 12:905–7. doi: 10.1038/nm1456 16862151

[24] Murphy BR Walsh EE. Formalin-inactivated respiratory syncytial virus vaccine induces antibodies to the fusion glycoprotein that are deficient in fusion-inhibiting activity. J Clin Microbiol (1988) 26:1595–7. doi: 10.1128/jcm.26.8.1595-1597.1988 PMC266671 2459154

[25] Prince GA Jenson AB Hemming VG Murphy BR Walsh EE Horswood RL. Enhancement of respiratory syncytial virus pulmonary pathology in cotton rats by prior intramuscular inoculation of formalin-inactiva ted virus. J Virol (1986) 57:721–8. doi: 10.1128/jvi.57.3.721-728.1986 PMC252798 2419587

[26] Delgado MF Coviello S Monsalvo AC Melendi GA Hernandez JZ Batalle JP. Lack of antibody affinity maturation due to poor toll-like receptor stimulation leads to enhanced respiratory syncytial virus disease. Nat Med (2009) 15:34–41. doi: 10.1038/nm.1894 19079256 PMC2987729

[27] Openshaw PJM Culley FJ Olszewska W. Immunopathogenesis of vaccine-enhanced RSV disease. Vaccine (2001) 20:S27–31. doi: 10.1016/S0264-410X(01)00301-2 11587806

[28] de Swart RL Kuiken T Timmerman HH van Amerongen G van den Hoogen BG Vos HW. Immunization of macaques with formalin-inactivated respiratory syncytial virus (RSV) induces interleukin-13-Associated hypersensitivity to subsequent RSV infection. J Virol (2002) 76:11561–9. doi: 10.1128/JVI.76.22.11561-11569.2002 PMC136757 12388717

[29] RSV Vaccine and mAb snapshot (2021). Available at: https://path.azureedge.net/media/documents/RSV-snapshot_28SEP2021_HighResolution.pdf (Accessed February 21, 2022).

[30] Sadoff J De Paepe E DeVincenzo J Gymnopoulou E Menten J Murray B. Prevention of respiratory syncytial virus infection in healthy adults by a single immunization of Ad26.RSV.preF in a human challenge study. J Infect Dis (2021). doi: 10.1093/infdis/jiab003 PMC9417128 33400792

[31] Salisch NC Izquierdo Gil A Czapska-Casey DN Vorthoren L Serroyen J Tolboom J. Adenovectors encoding RSV-f protein induce durable and mucosal immunity in macaques after two intramuscular administrations. NPJ Vaccines (2019) 4:54. doi: 10.1038/s41541-019-0150-4 31885877 PMC6925274

[32] Pierantoni A Esposito ML Ammendola V Napolitano F Grazioli F Abbate A. Mucosal delivery of a vectored RSV vaccine is safe and elicits protective immunity in rodents and nonhuman primates. Mol Ther - Methods Clin Dev (2015) 2:15018. doi: 10.1038/mtm.2015.18 26015988 PMC4441047

[33] Kohlmann R Schwannecke S Tippler B Ternette N Temchura VV Tenbusch M. Protective efficacy and immunogenicity of an adenoviral vector vaccine encoding the codon-optimized f protein of respiratory syncytial virus. J Virol (2009) 83:12601–10. doi: 10.1128/JVI.01036-09 PMC2786764 19776123

[34] He T-C Zhou S da Costa LT Yu J Kinzler KW Vogelstein B. A simplified system for generating recombinant adenoviruses. Proc Natl Acad Sci (1998) 95:2509–14. doi: 10.1073/pnas.95.5.2509 PMC19394 9482916

[35] Reed LJ Muench H. A simple method of estimating fifty per cent endpoints. Am J Hyg (1938) 27:493–7.

[36] Lapuente D Stab V Storcksdieck genannt Bonsmann M Maaske A Köster M Xiao H. Innate signalling molecules as genetic adjuvants do not alter the efficacy of a DNA-based influenza a vaccine. PLoS One (2020) 15:e0231138. doi: 10.1371/journal.pone.0231138 32243477 PMC7122823

[37] Anderson KG Sung H Skon CN Lefrancois L Deisinger A Vezys V. Cutting edge: Intravascular staining redefines lung CD8 T cell responses. J Immunol (2012) 189:2702–6. doi: 10.4049/jimmunol.1201682 PMC3436991 22896631

[38] Powell K. The race to make vaccines for a dangerous respiratory virus. Nature (2021) 600:379–80. doi: 10.1038/d41586-021-03704-y 34893769

[39] Pober JS Sessa WC. Evolving functions of endothelial cells in inflammation. Nat Rev Immunol (2007) 7:803–15. doi: 10.1038/nri2171 17893694

[40] Dinarello CA. Immunological and inflammatory functions of the interleukin-1 family. Annu Rev Immunol (2009) 27:519–50. doi: 10.1146/annurev.immunol.021908.132612 19302047

[41] Ben-Sasson SZ Hu-Li J Quiel J Cauchetaux S Ratner M Shapira I. IL-1 acts directly on CD4 T cells to enhance their antigen-driven expansion and differentiation. Proc Natl Acad Sci U S A (2009) 106:7119–24. doi: 10.1073/pnas.0902745106 PMC2678417 19359475

[42] Ben-Sasson SZ Hogg A Hu-Li J Wingfield P Chen X Crank M. IL-1 enhances expansion, effector function, tissue localization, and memory response of antigen-specific CD8 T cells. J Exp Med (2013) 210:491–502. doi: 10.1084/jem.20122006 23460726 PMC3600912

[43] Pang IK Ichinohe T Iwasaki A. IL-1R signaling in dendritic cells replaces pattern-recognition receptors in promoting CD8^+^ T cell responses to influenza a virus. Nat Immunol (2013) 14:246–53. doi: 10.1038/ni.2514 PMC3577947 23314004

[44] Kayamuro H Yoshioka Y Abe Y Arita S Katayama K Nomura T. Interleukin-1 family cytokines as mucosal vaccine adjuvants for induction of protective immunity against influenza virus. J Virol (2010) 84:12703–12. doi: 10.1128/JVI.01182-10 PMC3004317 20881038

[45] McCune C. Interleukin-1 as an adjuvant for tumor vaccines increases survival in mice. Biotherapy (1989) 1:355–9. doi: 10.1007/BF02171011 2701649

[46] Van Den Eeckhout B Van Hoecke L Burg E Van Lint S Peelman F Kley N. Specific targeting of IL-1β activity to CD8+ T cells allows for safe use as a vaccine adjuvant. NPJ Vaccines (2020) 5:64. doi: 10.1038/s41541-020-00211-5 32714571 PMC7378068

[47] Van Den Eeckhout B Huyghe L Van Lint S Burg E Plaisance S Peelman F. Selective IL-1 activity on CD8 + T cells empowers antitumor immunity and synergizes with neovasculature-targeted TNF for full tumor eradication. J Immunother Cancer (2021) 9:e003293. doi: 10.1136/jitc-2021-003293 34772757 PMC8593706

[48] Goritzka M Makris S Kausar F Durant LR Pereira C Kumagai Y. Alveolar macrophage–derived type I interferons orchestrate innate immunity to RSV through recruitment of antiviral monocytes. J Exp Med (2015) 212:699–714. doi: 10.1084/jem.20140825 25897172 PMC4419339

[49] Bhoj VG Sun Q Bhoj EJ Somers C Chen X Torres J-P. MAVS and MyD88 are essential for innate immunity but not cytotoxic T lymphocyte response against respiratory syncytial virus. Proc Natl Acad Sci (2008) 105:14046–51. doi: 10.1073/pnas.0804717105 PMC2532974 18780793

[50] Kirsebom FCM Kausar F Nuriev R Makris S Johansson C. Neutrophil recruitment and activation are differentially dependent on MyD88/TRIF and MAVS signaling during RSV infection. Mucosal Immunol (2019) 12:1244–55. doi: 10.1038/s41385-019-0190-0 PMC6778055 31358860

[51] Kandasamy M Suryawanshi A Tundup S Perez JT Schmolke M Manicassamy S. RIG-I signaling is critical for efficient polyfunctional T cell responses during influenza virus infection. PLoS Pathog (2016) 12:e1005754. doi: 10.1371/journal.ppat.1005754 27438481 PMC4954706

[52] Luo H Winkelmann E Xie G Fang R Peng B-H Li L. MAVS is essential for primary CD4 + T cell immunity but not for recall T cell responses following an attenuated West Nile virus infection. J Virol (2017) 91. doi: 10.1128/JVI.02097-16 PMC5331791 28077630

[53] Luke JM Simon GG Soderholm J Errett JS August JT Gale M. Coexpressed RIG-I agonist enhances humoral immune response to influenza virus DNA vaccine. J Virol (2011) 85:1370–83. doi: 10.1128/JVI.01250-10 PMC3020507 21106745

[54] Kulkarni RR Rasheed MAU Bhaumik SK Ranjan P Cao W Davis C. Activation of the RIG-I pathway during influenza vaccination enhances the germinal center reaction, promotes T follicular helper cell induction, and provides a dose-sparing effect and protective immunity. J Virol (2014) 88:13990–4001. doi: 10.1128/JVI.02273-14 PMC4249139 25253340

[55] Paulsen M Varese A Pinpathomrat N Kirsebom FCM Paulsen M Johansson C. MAVS deficiency is associated with a reduced T cell response upon secondary RSV infection in mice. Front Immunol (2020) 11:572747. doi: 10.3389/fimmu.2020.572747 33123150 PMC7573121

[56] Varese A Nakawesi J Farias A Kirsebom FCM Paulsen M Nuriev R. Type I interferons and MAVS signaling are necessary for tissue resident memory CD8+ T cell responses to RSV infection. PLoS Pathog (2022) 18:e1010272. doi: 10.1371/journal.ppat.1010272 35108347 PMC8843175

[57] Dickow J Francois S Kaiserling R-L Malyshkina A Drexler I Westendorf AM. Diverse immunomodulatory effects of individual IFNα subtypes on virus-specific CD8+ T cell responses. Front Immunol (2019) 10:2255. doi: 10.3389/fimmu.2019.02255 31608062 PMC6771563

[58] Wertz GW Collins PL Huang Y Gruber C Levine S Ball LA. Nucleotide sequence of the G protein gene of human respiratory syncytial virus reveals an unusual type of viral membrane protein. Proc Natl Acad Sci (1985) 82:4075–9. doi: 10.1073/pnas.82.12.4075 PMC397937 3858865

[59] Kishko M Catalan J Swanson K DiNapoli J Wei C-J Delagrave S. Evaluation of the respiratory syncytial virus G-directed neutralizing antibody response in the human airway epithelial cell model. Virology (2020) 550:21–6. doi: 10.1016/j.virol.2020.08.006 32866728

[60] Zohar T Hsiao JC Mehta N Das J Devadhasan A Karpinski W. Upper and lower respiratory tract correlates of protection against respiratory syncytial virus following vaccination of nonhuman primates. Cell Host Microbe (2022) 30:41–52.e5. doi: 10.1016/j.chom.2021.11.006 34879230

[61] Omokanye A Ong LC Lebrero-Fernandez C Bernasconi V Schön K Strömberg A. Clonotypic analysis of protective influenza M2e-specific lung resident Th17 memory cells reveals extensive functional diversity. Mucosal Immunol (2022). doi: 10.1038/s41385-022-00497-9 PMC8903128 35260804

[62] Schmidt ME Knudson CJ Hartwig SM Pewe LL Meyerholz DK Langlois RA. Memory CD8 T cells mediate severe immunopathology following respiratory syncytial virus infection. PLoS Pathog (2018) 14:e1006810. doi: 10.1371/journal.ppat.1006810 29293660 PMC5766251

[63] Schmidt ME Meyerholz DK Varga SM. Pre-existing neutralizing antibodies prevent CD8 T cell-mediated immunopathology following respiratory syncytial virus infection. Mucosal Immunol (2020) 13:507–17. doi: 10.1038/s41385-019-0243-4 PMC7181396 31844172

[64] Floc’h A Jalil A Franciszkiewicz K Validire P Vergnon I Mami-Chouaib F. Minimal engagement of CD103 on cytotoxic T lymphocytes with an e-Cadherin-Fc molecule triggers lytic granule polarization *via* a phospholipase cγ–dependent pathway. Cancer Res (2011) 71:328–38. doi: 10.1158/0008-5472.CAN-10-2457 21224355

[65] Altamirano-Lagos MJ Díaz FE Mansilla MA Rivera-Pérez D Soto D McGill JL. Current animal models for understanding the pathology caused by the respiratory syncytial virus. Front Microbiol (2019) 10:873. doi: 10.3389/fmicb.2019.00873 31130923 PMC6510261

[66] Graham BS Perkins MD Wright PF Karzon DT. Primary respiratory syncytial virus infection in mice. J Med Virol (1988) 26:153–62. doi: 10.1002/jmv.1890260207 3183639

