# Supplementary material for: Mucosal immunization with an adenoviral vector vaccine confers superior protection against RSV compared to natural immunity
Source: Front Immunol. 2022 Jul 28;13:920256. doi: 10.3389/fimmu.2022.920256 (PMC9394428; doi:10.3389/fimmu.2022.920256)
Supplement: Supplementary Figure 1 — Bioactivity of genetic adjuvants. The activation of NFκB- (A), ISRE- (B), IRF3- (C) and IFN-β- (D) pathways were assessed by in vitro luciferase reporter assays. To this end, HEK 293T cells were transfected with respective luciferase reporter plasmid followed by a transduction with Ad-empty, Ad-IPS-1 or Ad-IL-1β. Transfected cells without transduction were used as negative control (no Ad). Data represent medians of technical triplicates with individual data points. Data were analyzed by one-way ANOVA followed by Tukey´s post test (values were log-transformed before analysis). *p<0.05, ** p<0.005, *** p<0.0005, **** p<0.0001. Relative light units per second, RLU/s. [file DataSheet_1.pdf]

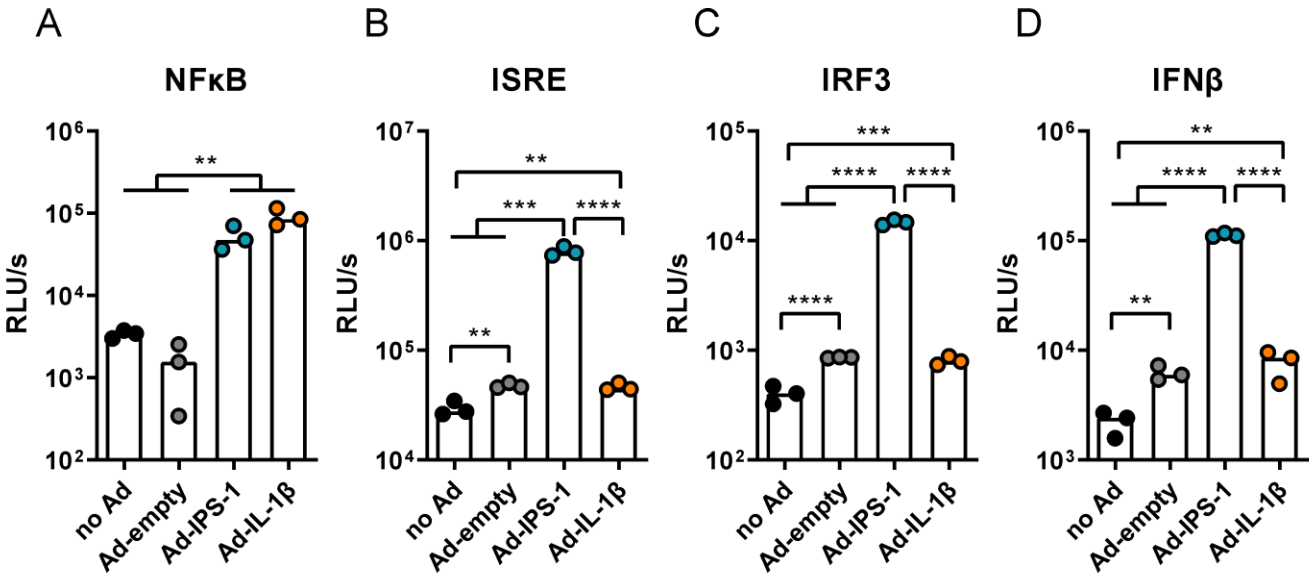

Supplemental Fig 1: Bioactivity of genetic adjuvants. The activation of NFκB- (A), ISRE- (B), IRF3- (C) and IFN-β- (D) pathways were assessed by in vitro luciferase reporter assays. To this end, HEK 293T cells were transfected with respective luciferase reporter plasmid followed by a transduction with Ad-empty, Ad-IPS-1 or Ad-IL-1β. Transfected cells without transduction were used as negative control (no Ad). Data represent medians of technical triplicates with individual data points. Data were analyzed by one-way ANOVA followed by Tukey´s post test (values were log-transformed before analysis). *$p<0.05$, ** $p<0.005$, *** $p<0.0005$, **** $p<0.0001$. Relative light units per second, RLU/s.

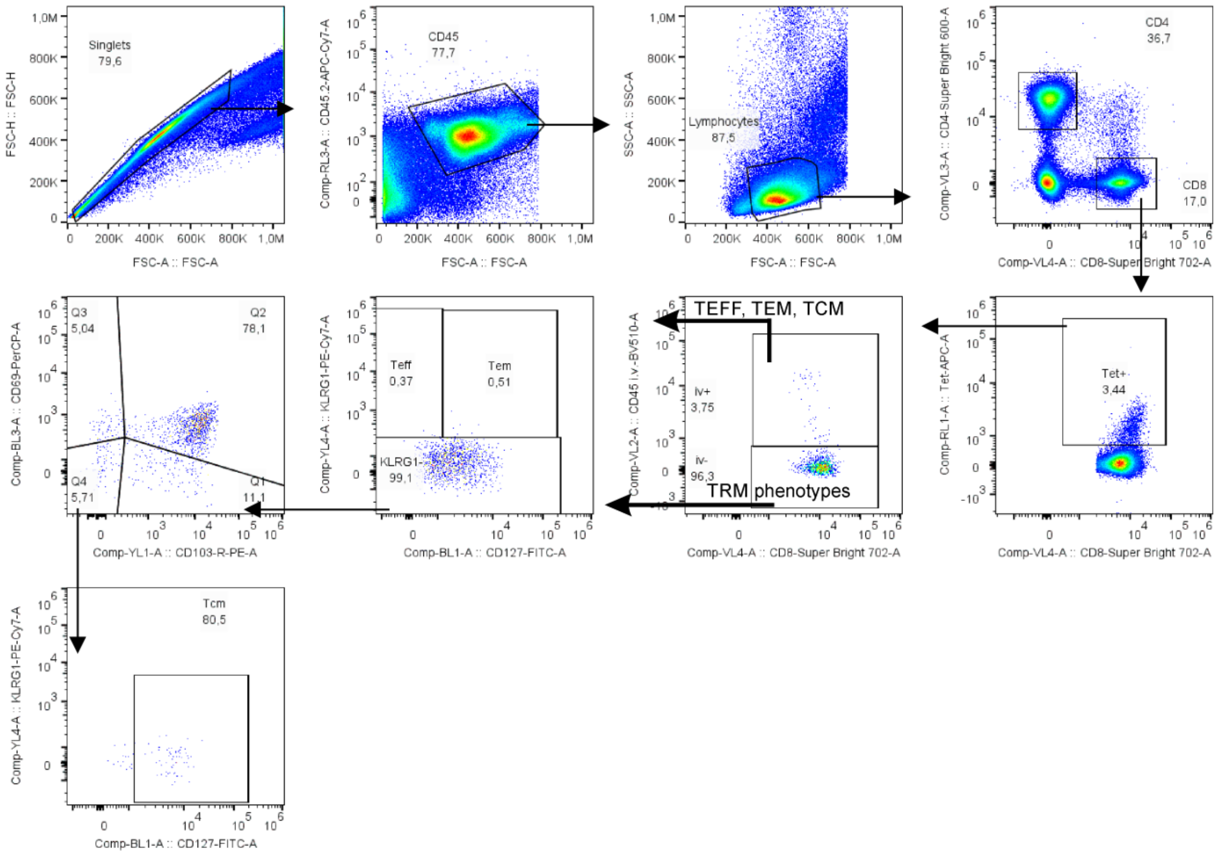

Supplemental Figure 2: Gating strategy for memory T cell phenotypes. Exemplary gating strategy of a lung sample from an Ad-F+Ad-IL-1β-immunized mouse. T_{RM} phenotypes are determined within the CD45-BV510 (intravascular staining) negative compartment, while circulatory T_{EFF}, T_{EM}, and T_{CM} are identified within the iv-positive compartment. T_{EFF}, effector T cells; T_{EM}, effector memory T cells; T_{CM}, central memory T cells; T_{RM}, tissue-resident memory T cells

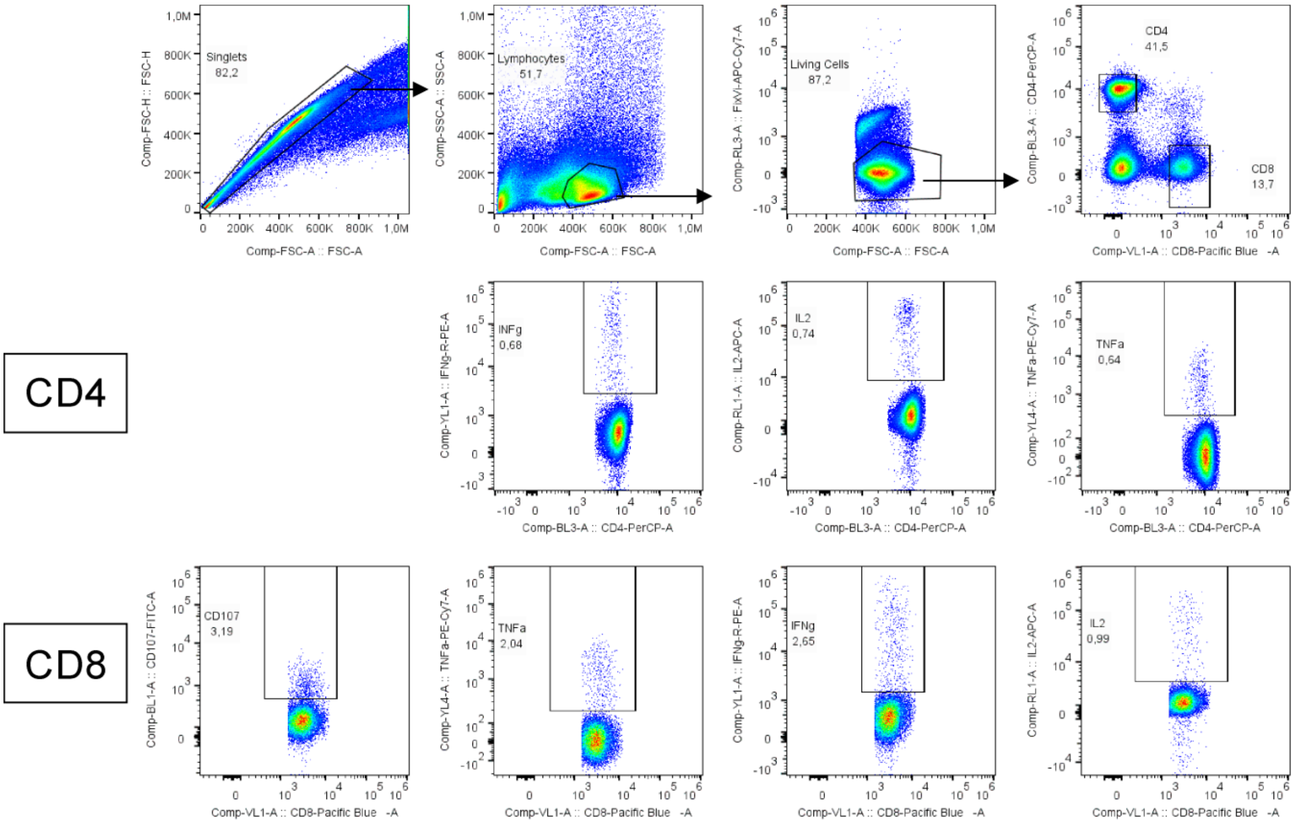

Supplemental Figure 3: Gating strategy for the analysis of functional T cell responses. Exemplary gating strategy of a lung sample from an Ad-F+Ad-IL-1β-immunized mouse. The degranulation marker CD107a was only assessed in $CD8^+$ T cells.

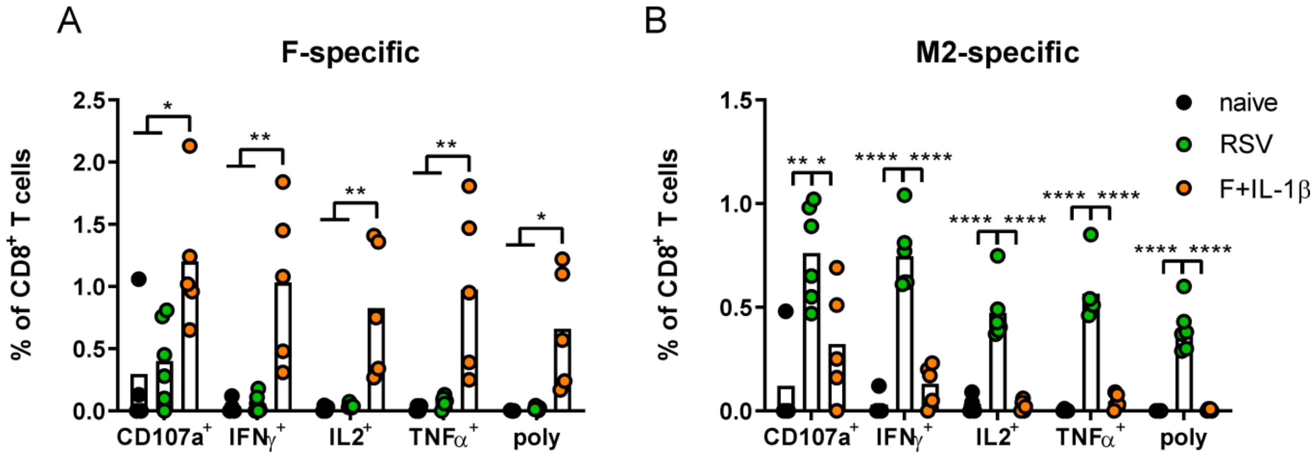

Supplemental Figure 4: Spleen T cell functionality induced by mucosal vaccination and a primary RSV infection. Spleen lymphocytes were restimulated with MHC-class I peptides derived from the F (A) and the M2 protein (B) and the functionality of $CD8^+$ T cells was detected via extracellular staining for CD107a and intracellular cytokine staining (poly; for $CD8^+$: $CD107a^+IFN\gamma^+IL\text{-}2^+TNF\alpha^+$; gating strategy in Supplemental Figure 3). Bars represent group means overlaid with individual data points. n=4-6. Data were analyzed by one-way ANOVA followed by Tukey´s post Test. *$p<0.05$, ** $p<0.005$, *** $p<0.0005$, **** $p<0.0001$.
